# Giant and tunable optical torque for micro-motors by increased force arm and resonantly enhanced force

**DOI:** 10.1038/s41598-018-21235-x

**Published:** 2018-02-12

**Authors:** Yong Geng, Jiubin Tan, Yongyin Cao, Yixuan Zhao, Zhengjun Liu, Weiqiang Ding

**Affiliations:** 1 Center of Ultra-Precision Optoelectronic, Instrument Engineering, Harbin Institute of Technology, Harbin, 150001 China; 2 Physics Department, Harbin Institute of Technology, Harbin, 150001 China; 3 School of Electro-Optical Engineering, Changchun University of Science and Technology, Changchun, 130022 China

## Abstract

Micro-motors driven by light field have attracted much attentions for their potential applications. In order to drive the rotation of a micro-motor, structured optical beams with orbital angular momentum, spin angular momentum, anisotropic medium, and/or inhomogeneous intensity distribution should be used. Even though, it is still challenge to increase the optical torques (OT) in a flexible and controllable way in case of moderate incident power. In this paper, a new scheme achieving giant optical torque is proposed by increasing both the force arm and the force amplitude with the assistance of a ring resonator. In this case, the optical torque doesn’t act on the target directly by the incident beam, but is transmitted to it by rotating the ring resonator connected with it. Using the finite-difference in time-domain method, we calculate the optical torque and find that both the direction and the amplitude of the torque can be tuned flexibly by modifying the frequency, or the relative phases of the sources. More importantly, the optical torque obtained here by linearly polarized beams can be 3 orders larger than those obtained using the structured beams. This opt-mechanical-resonator based optical torque engineering system may find potential applications in optical driven micro-machines.

## Introduction

Light with both linear and angular momenta can interact with objects and transfer the momenta to them, which results in optical forces (OFs) and optical torques (OTs)^1–3^. Mainly using the optical linear momentum^4^, stable optical trapping^5,6^, long distance transportation (both pushing and pulling)^7–10^, and optical cooling^11–13^ have been extensively investigated in various disciplines. At the same time, the angular momentum carried by photons can also induce a mechanical torque via scattering or absorbance, which also has countless applications. For example, in biology and biotechnology, OT has been used to rotate living cells and achieve motor proteins^14,15^. Using the OT, microscopic machines^16–18^, and integrated light devices^19–25^ with determined functionalities have also been demonstrated.

**Figure 1:**
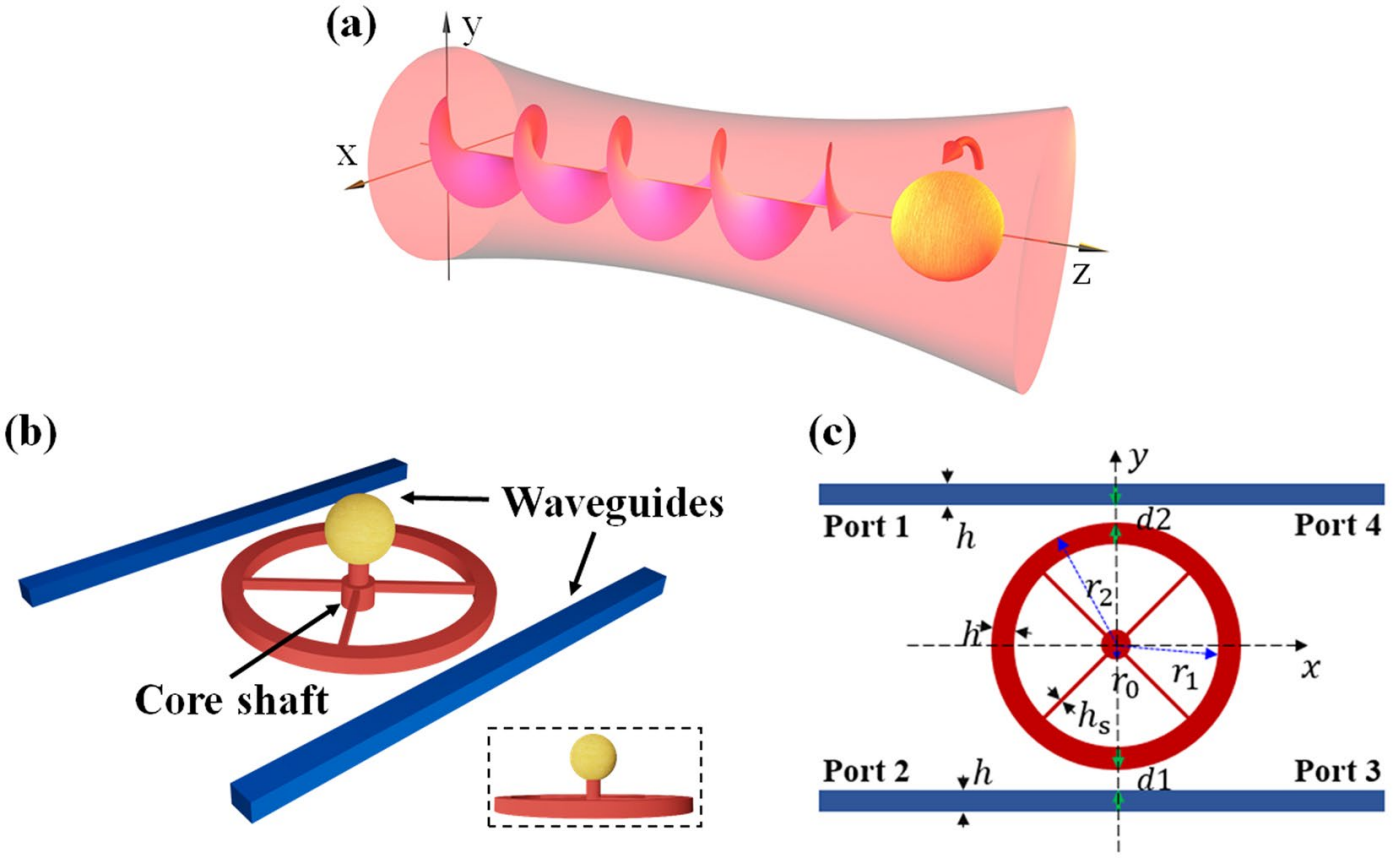
Schematic diagram of the giant and tunable optical torque on an object with the assistance of a ring resonator. (**a**) Traditional optical torque generation on the object by direct illumination with a structured light beam carrying angular or spin momentum. (**b**) Giant optical torque generation on the object by rotating the optical ring resonator (the red part), which mainly includes two parts: the optical part (the ring resonator and two feeding waveguides) and the mechanical part (the target and the connector, i.e., the sphere and the cores shaft, respectively). The ring resonator is feed through two fixed bus waveguides (the blue part) with linear polarization sources. Through the four connecting spokes, the ring resonator can rotate smoothly around the core shaft when driven by the optical torque. Owing to connecting rigidly with the connector (the rigid core shaft), the target (the gold sphere) will rotate synchronously with the connector at the same angular velocity. Suppose the bus waveguides, motor resonator, spokes and shaft are all made of silicon (refractive index of 3.4). (**c**) Two dimensional (2D) projection of the structure on the *xy*-plane, which is used in the numerical investigation of this paper. The ring resonator and the core shaft both centered at the coordination origin of (0,0). The parameters used in the simulations are *r*_0_ = 320 nm, *r*_1_ = 2.2 μm, *r*_2_ = 2.4 μm, *h* = 0.2 μm, *h*_*s*_ = 54 nm, *d*_1_ and *d*_2_ are the separation between the waveguides and ring *d*_1_ + *d*_2_ = 0.4 μm. With these parameters, the resonant wavelength of the ring resonator is near the communication wavelength 1550 nm.

Generally speaking, there are two common methods to excite OT. The first one is using light beams with orbital and/or spin angular momentum^26,27^, as demonstrated by Allen *et al*., as shown in Fig. 1(a). In this case, the light beams with a helical wave front defined by the azimuthal angle *θ* of $\exp (im\theta )$ (*m* is the topology charge, an integer) possess an amount of orbital angular momentum (OAM) of *mħ* per photon. On the other hand, the circularly polarized beams possess an amount of spin angular momentum (SAM) of ±*ħ* per photon. Both the OAM and SAM can be transferred to objects (which may be symmetric and isotropic) and generate an OT^28–30^. The second one is using anisotropic or chiral medium, or asymmetric structures^31–34^. In this case, no structured beams with OAM or SAM are necessary, and OT is mainly generated by the asymmetrical distribution of the OFs inside the object^35,36^.

Even though the OT has been demonstrated by Beth about 80 years ago^1^, and has been intensively investigated since Allen’s work^26,27^, it’s still not effortless to enhance and adjust the OT simultaneously and arbitrarily. This restricts the applications in micro-motor and related opt-mechanical technologies. For example, in order to increase the orbital torque, one has to increase the topological charge *m* of the vortex beams, which is challenging in practice, and it also has intrinsic effect to the beams’ shape. For the spin torque, one has less chances to increase it since only two circular polarization states are available.

In order to increase the OT significantly and tune it flexibly, here, we propose a new scheme to generate a giant OT which can drive a micro-motor efficiently. Since the torque is determined simultaneously by the optical force amplitude and force arm, both of them are enhanced in our scheme with the utilization of a ring resonator, as shown in Fig. 1(b) and (c). In this scheme, the OT is not act on the target directly as usual, but is transmitted to it through the connected optical ring resonator, which is rotated by linearly polarized sources. The merits of this configuration lie in the following aspects. First, the force arm can be increased efficiently by the radius of the ring resonator but not limited by the light beam nor the target object. Second, the force could be enhanced and tuned flexibly by selecting the incident frequency detunes from the resonance center of the ring. Third, since the light does not illuminate on the objects directly, it is free from optical damage when the incident power is increased to an ultrahigh level. Fourth, no structured angular momentum source is needed, and only linear polarization sources are used. Results show that the OTs reported here can be 3 orders larger than those obtained using the orbital and spin angular momentums of light at the same incident level. Different from those OTs on levitated objects^19,37–39^, our model works with a fixed rotation shaft which ensures a stronger stability and can be applied to integrated optics more conveniently.

## Results and Discussions

### Structure design and simulation configurations

The schematic structure investigated here is shown in Fig. 1(b) and (c). For comparison, Fig. 1(a) shows the traditional method to generate an OT, where the target object is illuminated by a structured light beam with angular momentum. In this case, the object and the beam cross section should be matched in size in order to transfer the angular momentum efficiently. Figure 1(b) shows the three dimensional (3D) schematic diagram to generate a giant and tunable OT. It includes two main parts: The first one is the optical part formed by the ring resonator and two feeding waveguides and the second is the mechanical part consisting of the target and the connector (i.e., the sphere and the core shaft, respectively). The light source is imported into the system through one or more ports of waveguides (blue part) and coupled into the ring resonator. Then the ring resonator can rotate around the core shaft driven by optical torque. Suppose the target, which is a sphere in current situation and also can be any other complex micro-motor (not shown in this figure), is connected rigidly to the connector (the rigid core shaft), as shown in Fig. 1(b), it will rotate synchronously with the connector at the same angular velocity. Ultimately, the target is driven by the input light indirectly (through the ring resonator and the connector), which is different from the mechanism of direct illuminating by light, shown in Fig. 1(a).

From the analysis above, we can see that the target object, the connector, and the ring resonator are connected with each other rigidly, and can rotate synchronously. Thus they can be regarded as a whole entirety. From this point of view, we regard that the optical torque on the ring resonator is transferred to the target object without loss. Due to this reason, we only focus on the optical torque investigation on the ring in this work. We believe that this lossless transfer of torque can be realized in practice (either exactly, or approximately at least), provided the connector is a rigid one.

For convenience, the four ports are named 1, 2, 3 and 4, respectively. One or two of the ports will be excited in order to tune the OTs. Here, it should be noted that the excitation of some port means that a light source is injected into the waveguide from this port. The two dimensional (2D) projection of the structure is shown in Fig. 1(c), which is the model used in numerical simulation. The ring resonator shown in Fig. 1(c) with a core shaft (red, at the original center of (0,0)) and bus waveguides (blue) are all made of silicon with a refractive index of *n*_WG_ = 3.4, and thickness of *h* = 0.2 μm (The thicknesses of the two bus waveguides and the ring resonator are the same). The radius of the small core shaft is *r*_0_ = 320 nm, and the inner and outer radiuses of the ring resonator are *r*_1_ = 2.2 μm, *r*_2_ = 2.4 μm, respectively. The two bus waveguides are fixed on the substrate, while the ring resonator restricted the core shaft can rotate smoothly in the xy-plane when driven by the OT. The whole system is embedded in water with a refractive index of *n*_BG_ = 1.33.

It is noted that, in this work, the light source is feed into the system through the waveguides, and is confined into the waveguides and the ring resonator very well since is propagates in them as a guiding mode. Thus, the target object and the connector (the core shaft) do not affect significantly the light propagation inside the waveguide and the ring resonator. Based on this fact, the two dimensional structure, as shown in Fig. 1(c), is used in the simulation, but all the results and conclusions presented here can be extended to 3D straightforwardly. On the other hand, due to the similarity of the TE and TM modes in this 2D structure, only the results for TM polarization (with *H*_*x*_, *H*_*y*_ and *E*_*z*_ nonzero) are shown.

The simulation method used in this work is the finite-difference in time-domain (FDTD) method with well tested accuracy and convergence. When all the electromagnetic fields are extracted from the simulation programs, the time averaged OT 〈**Γ**〉 exerted on the ring resonator is calculated using the formula of^40^.

$$\langle {\boldsymbol{\Gamma }}\rangle =-{\oint }_{L}\hat{{\bf{n}}}\cdot \langle \overleftrightarrow{{\bf{T}}}\rangle \times {\bf{r}}d{\bf{l}}$$

Here, $\overleftrightarrow{{\bf{T}}}$ is the Maxwell’s stress tensor, and *L* is the integral contour loop surrounding the ring, as denoted in Fig. 1(c), **r** is the position vector of the integral contour related to the ring center, and $\hat{{\bf{n}}}$ is the unit outer normal vector of the contour *L*. Please refer the section of Methods for more details used in the simulation.

### Giant optical torque at off resonant wavelengths

**Figure 2:**
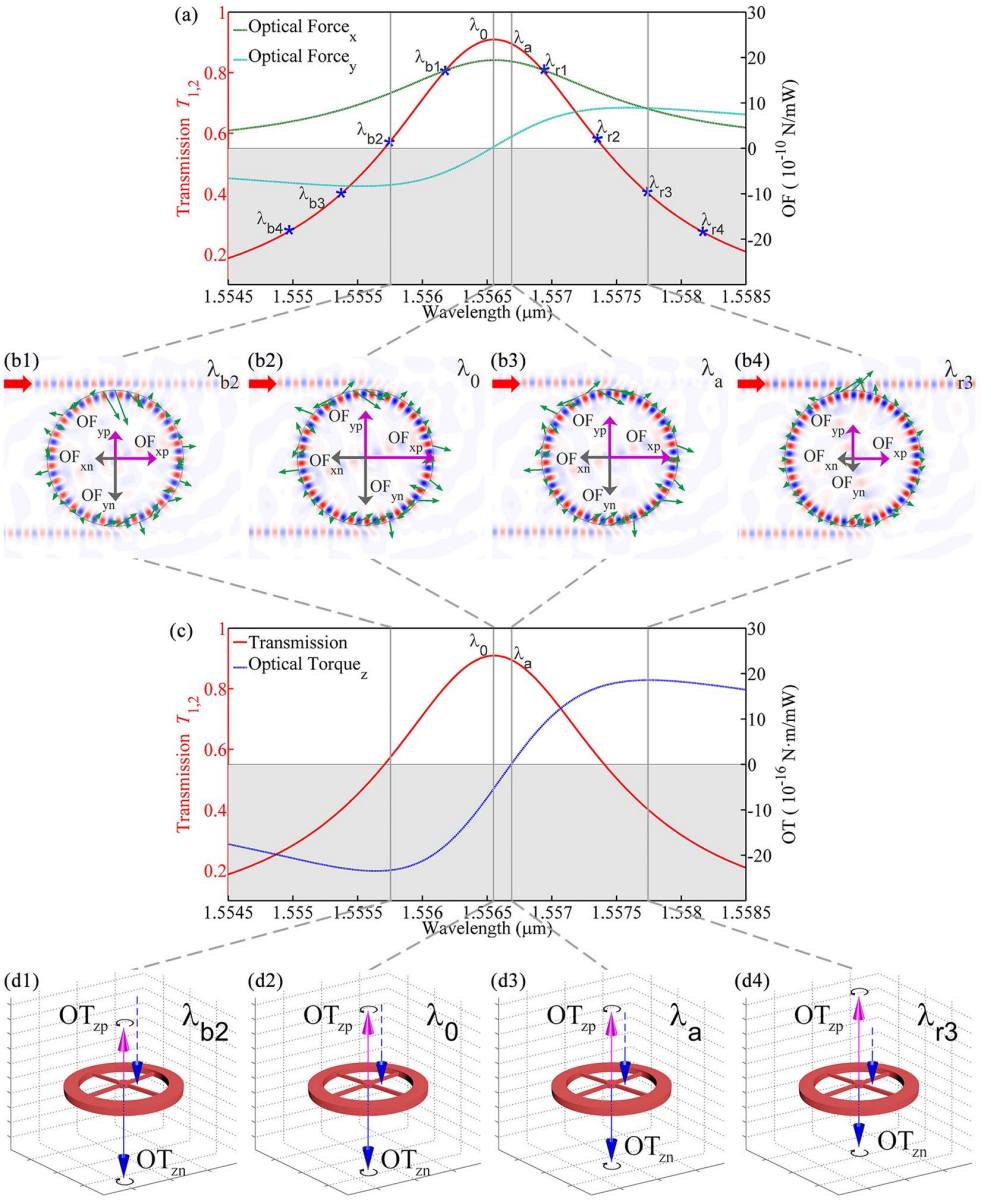
Optical force and torque on the ring resonator when it is located at (0,0), and only port 1 is excited. (**a**) Transmission spectrum *T*_1,2_ of the system (left *y*-axis, red solid curve) and the *x*, *y* components of the optical forces (right *y*-axis, green color for *x* component, and cyan color for *y* component) exerted on the ring resonator. The four gray vertical solid lines represent four typical wavelengths *λ*_b2_, *λ*_0_, *λ*_*a*_, *λ*_*r*3_ (resonant wavelength is *λ*_0_) and the eight blue star labels represent different extent excitation wavelengths that *λ*_*b*1–*b*4_(*λ*_*r*1–*r*4_) are the blue (red) detuned wavelength, which are analyzed detailedly in the following sections. (**b1**~**b4**) Distribution of electric field *E*_*z*_ and optical force density vector on the integral path (green arrows) overlapped by the positive and negative components of the optical forces in *x* and *y* directions, respectively (denoted by OF_xp_, OF_xn_, OF_yp_ and OF_yn_, respectively). (**c**) Optical torque exerted on the ring resonator (right *y*-axis, blue dotted curve). The transmission curve (left *y*-axis, red solid curve) is also drawn for reference. Here, negative (positive) OT means clockwise (anti-clockwise) rotation of the ring resonator. (**d1**–**d4**) Relative values of the positive and negative components of OT. For comparison, the blue arrows are also shown next to the red ones.

In the ring resonator structure, investigations showed that the optical force is strongly dependent on the detune of the source wavelength related to the resonant center^41^. In order to select proper source frequency, the transmission spectrum from the drop port 2 is calculated when port 1 is excited only, as shown in Fig. 2(a) (left *y*-axis). The resonant central wavelength is at *λ*_0_ = 1.55655 μm. The transmission efficiency is slightly less than 1 is due to the scattering loss introduced by the four spokes. The scattering of the central shaft, however, can be neglected, since there is almost no overlap between the guiding modes and the shaft, which is also verified in our numerical simulations. The optical forces are also shown in Fig. 2(a) (right-y axis) for the analysis of optical torque.

Figure 2(c) shows the OT exerted on the ring resonator when port 1 is excited. The four gray solid vertical lines denote four different excitation wavelength *λ*_b2_, *λ*_0_, *λ*_*a*_, *λ*_*r*3_(*λ*_0_ is the resonant wavelength) which will be analyzed in detail. The direction of the OT is perpendicular to *xy*-plane (along the *z* direction). The upper region with Γ > 0 means the ring will rotate counter-clockwise, and the lower gray shaded region with Γ < 0 means clockwise rotation. Figure 2(c) shows that both the direction and the amplitude of the torque can be tuned by the detune of wavelength. One can also notice that the negative maximum OTs at *λ*_*b*2_ is as large as −25 × 10^−16^(pN ⋅ m/mW). Comparison with the OTs obtained in traditional method using spin angular momentum beams, the OT obtained here is giant. For example, in ref.^21^, the OT generated by the spin angular momentum is about 1000 pN ⋅ nm(about 10^−18^ N ⋅ m), which is 3 orders smaller than the torque shown in Fig. 2(c).

Figure 2(c) shows that the OT is zero at the wavelength of *λ*_*a*_, while the optical force is not zero (see Fig. 2(a)). In order to understand this feature, we plot one part of density vector of OF (green arrows) on the integral contour and the sum of four components (positive/negative of the optical force in the *x* and *y* direction, i.e., OF_xp_, OF_xn_, OF_yp_ and OF_yn_, respectively) of the optical force (pink and green arrows) at the original center of (0,0) with four different excitation wavelengths *λ*_b2_, *λ*_0_, *λ*_*a*_, and *λ*_*r*3_ as shown in Fig. 2(b1–b4), where the distribution of the electrical field *E*_*z*_ is shown as the background. Accordingly, the sum of the positive and negative components of the OTs are shown in Fig. 2(d1–d4). Comparing Fig. 2(b3) and (d3), one can find that after the cancellation of OF_xp_ + OF_xn_ and OF_yp_ + OF_yn_, the optical force still remains a very large value. However, at this case, the absolute values of positive and negative components of the OT are the same and cancel each other out, which results in the final OT being zero. On the other hand, at the wavelengths of *λ*_*r*3_ = 1.55772 μm and *λ*_*b*2_ = 1.55570 μm, the optical force does not reach the maximum, but the OT reaches its positive and negative maximums, respectively.

### Optical torques with double-port excitation

**Figure 3:**
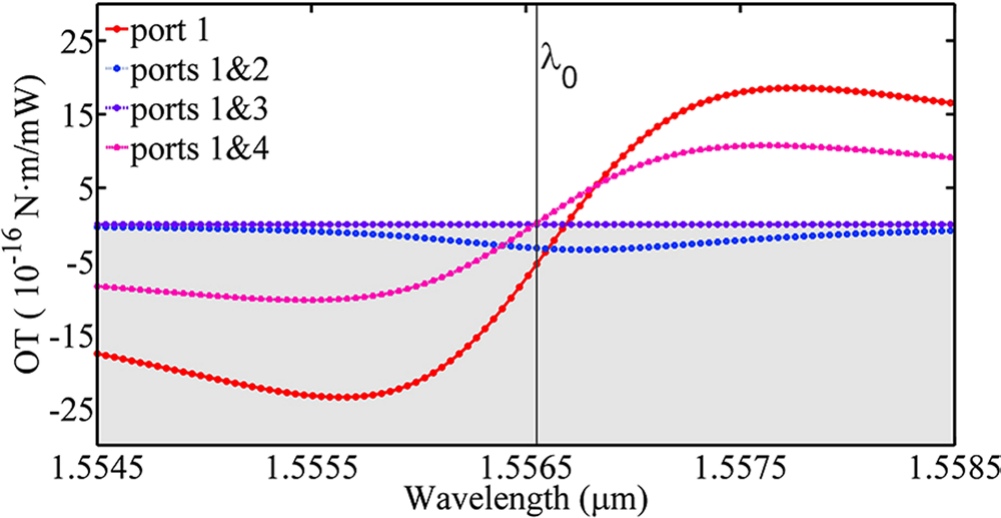
Optical torques with different double-port excitations.

It has been reported that the optical forces exerted on the ring can be tuned flexibly by the selected excitation of the ports^41^. Here, we find that the OT can also be tuned flexibly by the similar method. Figure 3 shows the different OTs at 3 various double-port excitation ways of ports 1 & 2 (blue, dashed curve), ports 1 & 3 (violet), ports 1 & 4 (pink), respectively. For comparison, the case of only port 1 (red, solid curve) excitation is also shown.

From Fig. 3, one can observe that the OTs with double-port excitation are smaller than the case single port excitation. Extreme example is the case of ports 1 & 4, where the torque is exactly zero. This is easy to understand since the two incident modes are collinear but with opposite propagation direction, which means that the total linear momentum and angular momentum of the incident beams are zero.

When ports 1 & 2 are excited simultaneously, the OT should also be zero since the total *angular momentum* of the two sources are zero due to the symmetry of ports 1 & 2 with respect to *x* axis, which is inconsistent with the blue dashed line shown in Fig. 3. The fact is, in order to quantify the effect of the asymmetrical of the ring resonator as the rotation occurs, that we designedly rotate the ring resonator to a maximal asymmetrical position, seen in Fig. 2(b1–b4). It turns out that the broken symmetry generated by the rotation of the ring resonator is insignificant.

When ports 1 & 3 are excited simultaneously, we have known that there is no optical force acting on the ring resonator^41^, which is due to the asymmetric Fano transmission^42,43^. Owing to the core shaft is at the point of (0,0), the geometric center of the whole model, it’s understandable that the OT (violet dotted curve in Fig. 3) remains zero with different excitation wavelengths.

**Figure 4:**
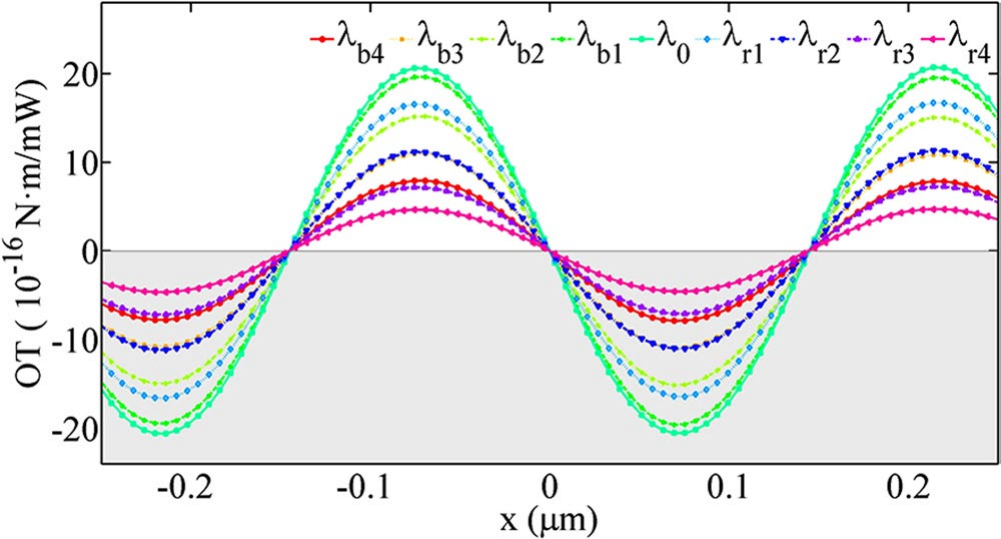
Optical torques versus the central position of the ring resonator when ports 1 & 3 are excited simultaneously with the same frequency and power. OT can be tuned sinusoidally with the position in this case.

Although the OTs are smaller in cases of double-port excitation, one advantage of double-port excitation is the tunability of the torque with the longitudinal position *x* of the ring. As shown above, when the incident wavelength is fixed, the OT cannot be tuned in case of single port excitation. When ports 1 & 3 are excited simultaneously with the fixed wavelength, however, the torque can be tuned sinusoidally with the central position *x* of the ring resonator, as shown in Fig. 4.

From Fig. 4, one knows that the OT, both the amplitude and direction, can be tuned by the position of the ring resonator. In practice, however, it’s inconvenient to change the position of the rotation shaft since it is connected with the substrate. Here, we find the solution to this disadvantage by tuning the relative phase difference Δ*θ*_13_ of the two excitation sources of ports of 1 & 3, which is equivalent to shift the ring resonator along *x* axis.

### Effect of transverse position on optical torque

**Figure 5:**
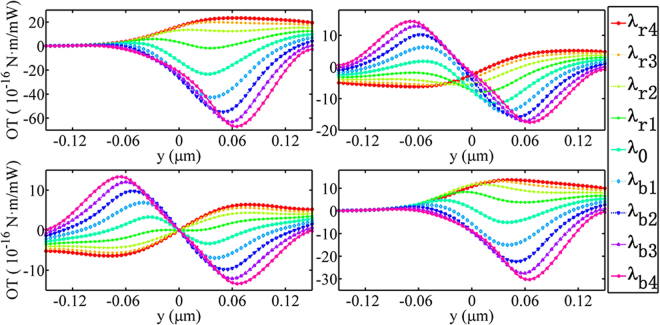
OT versus transverse position *y* for the four different excitation ways. (**a**) Port 1 is excited. (**b**) Ports 1 & 2 are excited. (**c**) Ports 1 & 3 are excited. (**d**) Ports 1 & 4 are excited.

In the analysis above, the ring resonator is supposed to be just on the middle of the two waveguides, i.e., *y* = 0. In practice, however, there may be a small deviation from the *y* = 0 unavoidably. Our results show that a small offset from the center does not affect the torque significantly, as shown in Fig. 5.

Figure 5 shows the OT versus the transverse shift of the ring resonator from *y* = 0. Figure 5(a) shows the results of port 1 excitation. When the core shaft shifts to the negative *y* direction, one can see that the OT decreases due to the coupling strength between the feeding waveguide and the ring becomes smaller and smaller. When the ring shifts to the positive direction, however, the amplitude of OT can be larger than 60 × 10^−16^ (pN ⋅ m/mW) at the case of *λ*_*b*4_ and *y* = 0.06 μm, which is larger than the case shown in Fig. 2. Similar results can be obtained when ports 1 & 4 are excited simultaneously, which are shown in Fig. 5(d). Figure 5(b) and (c) show the results of simultaneous excitation of ports 1 & 2 and ports 1 & 3, respectively. These results reveal that the direction and amplitude of the OT can also be adjusted by the transverse location of the ring resonator and excitation wavelength together.

### Effect of force arm on optical torque

**Figure 6:**
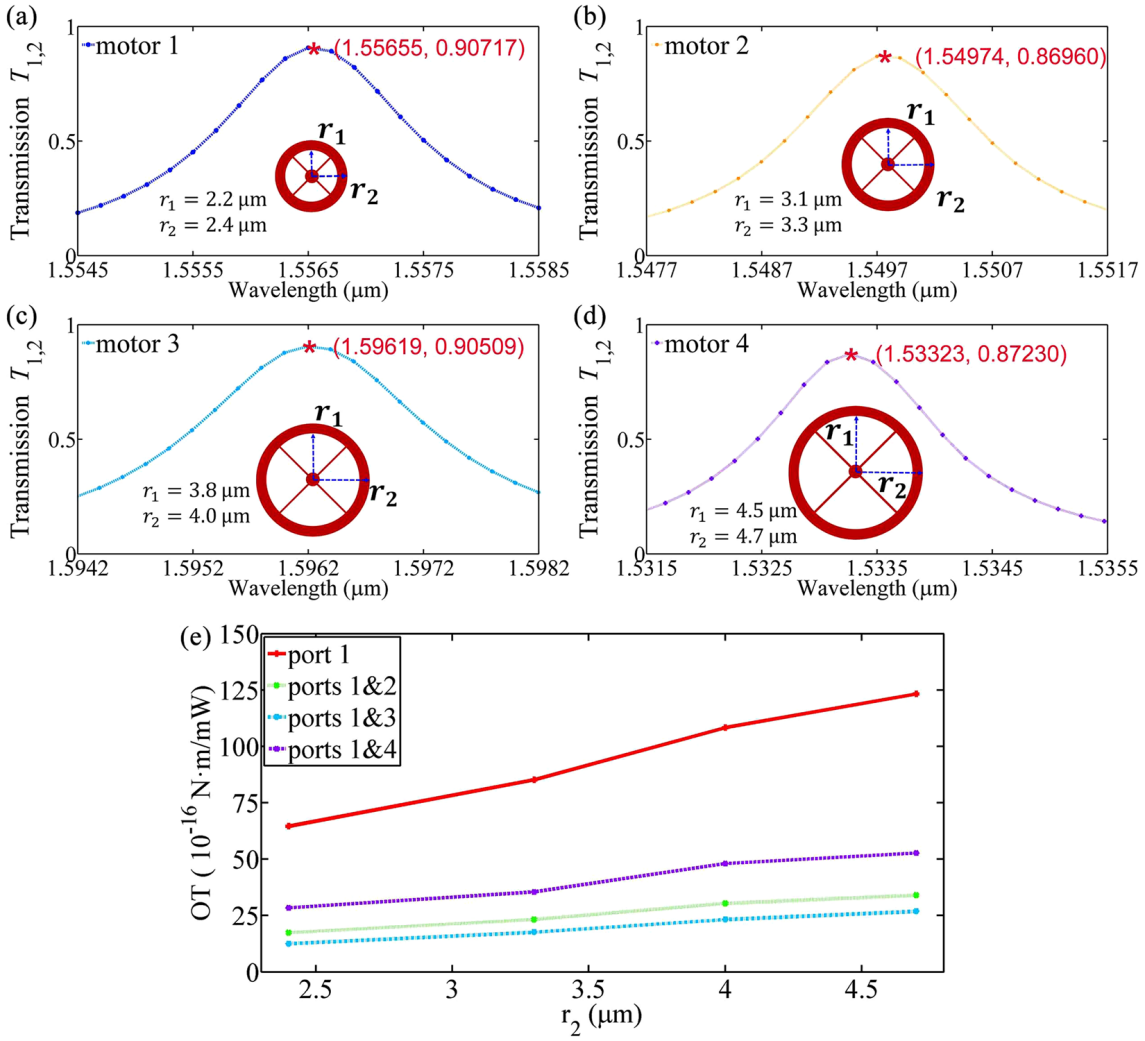
Optical torque versus the radius of the ring resonator. All other parameters, including the refractive index, width of the waveguide and ring, and the gap between them, are the same as those in Fig. 2(a–d). Transmission curve *T*_1,2_ of the four different ring resonators with radiuses of 2.4 μm, 3.3 μm, 4.0 μm and 4.7 μm, respectively, and the peak value coordinate are labeled by red stars, which show that the resonant wavelength *λ*_01~04_ are 1.55655 μm, 1.54974 μm, 1.59619 μm, 1.53323 μm and the peak transmission of motor 1~4 are 0.90717, 0.86960, 0.90509, 0.87230, respectively. (**b**) OT peak values changes with the radius of the ring resonator for different excitations.

As stated above, the force arm in current scheme can be very large and plays an important role for the giant OT. In order to demonstrate this point, we calculate four different radiuses of optical motors with different radiuses of 2.4 μm, 3.3 μm, 4.0 μm and 4.7 μm, respectively. For convenience, we name them motor 1 to motor 4, respectively, and the results are shown in Fig. 6. Figure 6(a–d) shows the transmission spectrum *T*_1,2_ of the motor 1~4. For a fair comparison, the wavelengths of *λ*_01_–*λ*_04_ with similar peak transmission coefficients are selected for the 4 motors in the OT calculation. When only port 1 is excited, and the optimized transverse positions *y* are selected, we calculate the absolute peak values of the OT, and results are shown in Fig. 6(e). From Fig. 6(e), we can observe that the OT is proportional to the radius of the ring, i.e., the force arm. For example, when the radius of the motor is increased from 2.4 μm to 4.7 μm, the amplitude of the OT acted on the motor is also doubled roughly. Compared to the popular way to generate an OT using the structured angular momentum beams, the mechanical adjustability of the force arm is another great advantage in our scheme.

## Discussions

The scheme of OT generation using simple guiding modes of linear polarization in a ring resonator has great advantages in practice. In previous research results, vector and/or vortex beams are popular ways to excite the OT, which are inefficient in order to increase the OT. Firstly, one has to generate a structured optical beam for the OT in the traditional method, which is not so easy, especially for a large topological charge. In our scheme, however, only simple guiding mode of linear polarization in a conventional waveguide is used, instead of complex vector and vortex beams. Secondly, since the light beam is incident on the object directly in traditional method, the high light intensity may damage the sample when the incident power is increased to a large level. In our new scheme, the light beam propagates in the ring resonator and the waveguides but not in the sample, thus it is free from optical damage. Thirdly, the OT generation efficiency is much higher in our new scheme than that in traditional method. In our scheme, the resonant coupling between the waveguide and the resonator increases the force significantly, and the large radius of the ring increases the force arm, which may be much larger than the dimension of the object. Those two factors result in the giant and tunable OT as demonstrated in the previous sections. In traditional method, the force arm is limited by the object size and the transverse extension of the light beam. Also, the scattering or absorption of the object to the structured beam is not flexible to control. Finally, we introduce the OT transfer in our scheme. On one end of the shaft, the ring is rotated by the incident beam, and a giant OT is generated. The other end of the shaft can couple with any kind of micro-motors, which makes current scheme broad potential applications.

## Conclusions

In conclusion, we have proposed a new scheme to achieve giant OT with the assistance of a ring resonator structure, which can increase both the force arm and the force amplitude simultaneously. In this scheme, only linear polarized optical modes supported in the ring resonator and the waveguides are used, which convert the linear momentum of the incident beams into OT through the asymmetrical force distribution on the resonator and the assistance of a supporting shaft. Results show that the OT is rather large comparing with those torques obtained in the popular method of using the orbital and/or spin angular momentum beams. This giant OT can provide a powerful engine for micro-motors, and may find potential applications in related technologies.

## Methods

In this paper, the FDTD method was adopted to calculate the coupled electromagnetic fields (**E**,**H**) between two parallel bus waveguides and the ring resonator, as shown in Fig. 1(b). The FDTD method discrete the Maxwell’s equations directly in temporal and spatial domains by utilizing difference quotient to replace partial derivative, and subsequently simulate the propagation of electromagnetic waves to get the field distributions. After the electromagnetic fields are obtained, the time averaged optical force and torque are able to be calculated by integrating the Maxwell’s stress tensor (MST) $\overleftrightarrow{{\bf{T}}}$ on a closed curve surrounding the ring resonator, as shown in Eqs (1) and (2)^41,44^.

1 $$\langle {\bf{F}}\rangle ={\oint }_{L}\langle \overleftrightarrow{{\bf{T}}}\rangle \cdot d{\bf{l}}$$

2 $$\langle {\boldsymbol{\Gamma }}\rangle =-{\oint }_{L}\hat{{\bf{n}}}\cdot \langle \overleftrightarrow{{\bf{T}}}\rangle \times {\bf{r}}d{\bf{l}}$$

Where *L* is the closed curve surrounding the ring resonator, $\overrightarrow{{\bf{r}}}$ is the position vector of any point on *L* with respect to the mass center of the ring resonator, and the MST is defined by $\overleftrightarrow{{\bf{T}}}={\bf{D}}\otimes {\bf{E}}+{\bf{B}}\otimes {\bf{H}}-\frac{1}{2}\overleftrightarrow{{\bf{I}}}({\bf{D}}\cdot {\bf{E}}+{\bf{B}}\cdot {\bf{H}})$, the average MST of a period about the total electric and magnetic fields (**E**, **H**) is expressed as

3 $$\langle \overleftrightarrow{{\bf{T}}}\rangle =\frac{1}{2}{\rm{R}}{\rm{e}}\{{\bf{D}}\otimes {{\bf{E}}}^{{\boldsymbol{\ast }}}+{\bf{B}}\otimes {{\bf{H}}}^{{\boldsymbol{\ast }}}-\frac{1}{2}\overleftrightarrow{{\bf{I}}}({\bf{D}}\cdot {{\bf{E}}}^{{\boldsymbol{\ast }}}+{\bf{B}}\cdot {{\bf{H}}}^{{\boldsymbol{\ast }}})\}$$

Where ⊗ is the dyadic operator, $\overleftrightarrow{{\bf{I}}}$ is the unit tensor of 3 × 3. From the OT, the light-driven behaviors of the ring resonator can be investigated. The Perfectly Matched Layer (PML) Boundary condition is set to the simulation boundaries, which aims to create a nonphysical absorber adjacent to the outer boundary that has a wave impedance independent of angle of incidence and frequency of outgoing scattered waves^45^.

## References

[1] Beth R (1936). Mechanical detection and measurement of the angular momentum of light. Physical Review A.

[2] Ashkin A (1970). Acceleration and trapping of particles by radiation pressure. Physical Review Letters.

[3] Friese MEJ, Enger J, RubinszteinDunlop H, Heckenberg NR (1996). Optical angular-momentum transfer to trapped absorbing particles. Physical Review A.

[4] Kajorndejnukul V, Ding WQ, Sukhov S, Qiu CW, Dogariu A (2013). Linear momentum increase and negative optical forces at dielectric interface. Nature Photonics.

[5] Grzegorczyk, T. M., Kemp, B. A. & Kong, J. A. Stable optical trapping based on optical binding forces. *Physical Review Letters* **96**10.1103/PhysRevLett.96.113903 (2006). 10.1103/PhysRevLett.96.113903 16605823

[6] De AK, Roy D, Dutta A, Goswami D (2009). Stable optical trapping of latex nanoparticles with ultrashort pulsed illumination. Appl. Optics.

[7] Intaraprasonk V, Fan S (2013). Optical pulling force and conveyor belt effect in resonator–waveguide system. Optics Letters.

[8] Cizmar T, Garces-Chavez V, Dholakia K, Zemanek P (2005). Optical conveyor belt for delivery of submicron objects. Applied Physics Letters.

[9] Schrader D (2001). An optical conveyor belt for single neutral atoms. Appl Phys B-Lasers O.

[10] Lu J (2017). Light-Induced Pulling and Pushing by the Synergic Effect of Optical Force and Photophoretic Force. Physical review letters.

[11] Wallace CD, Dinneen TP, Tan KYN, Grove TT, Gould PL (1992). Isotopic Difference in Trap Loss Collisions of Laser Cooled Rubidium Atoms. Physical Review Letters.

[12] Phillips WD, Prodan JV (1985). & Metcalf, H. J. Laser Cooling and Electromagnetic Trapping of Neutral Atoms. J Opt Soc Am B.

[13] Chu S, Wieman CL (1989). Cooling and Trapping of Atoms. J Opt Soc Am B.

[14] Gudipati M (2005). Optically-controllable, micron-sized motor based on live cells. Optics Express.

[15] Van den Heuvel MGL, Dekker C (2007). Motor proteins at work for nanotechnology. Science.

[16] Kuhn S (2017). Full rotational control of levitated silicon nanorods. Optica.

[17] Simpson SH, Hanna S (2010). Holographic optical trapping of microrods and nanowires. Journal of the Optical Society of America a-Optics Image Science and Vision.

[18] Shelton WA, Bonin KD, Walker TG (2005). Nonlinear motion of optically torqued nanorods. Phys. Rev. E.

[19] Liu M, Zentgraf T, Liu YM, Bartal G, Zhang X (2010). Light-driven nanoscale plasmonic motors. Nature Nanotechnology.

[20] Harada T, Yoshikawa K (2002). Mode switching of an optical motor. Applied Physics Letters.

[21] La Porta A, Wang MD (2004). Optical torque wrench: Angular trapping, rotation, and torque detection of quartz microparticles. Physical Review Letters.

[22] Kim K, Xu XB, Guo JH, Fan DL (2014). Ultrahigh-speed rotating nanoelectromechanical system devices assembled from nanoscale building blocks. Nat. Commun..

[23] Lin SY, Crozier KB (2012). An integrated microparticle sorting system based on near-field optical forces and a structural perturbation. Optics Express.

[24] Lin SY, Zhu WQ, Jin YH, Crozier KB (2013). Surface-Enhanced Raman Scattering with Ag Nanoparticles Optically Trapped by a Photonic Crystal Cavity. Nano Lett..

[25] Lin SY, Crozier KB (2013). Trapping-Assisted Sensing of Particles and Proteins Using On-Chip Optical Microcavities. ACS Nano.

[26] Babiker M, Power WL, Allen L (1994). Light-Induced Torque on Moving Atoms. Physical Review Letters.

[27] Allen L, Beijersbergen MW, Spreeuw RJC, Woerdman JP (1992). Orbital Angular-Momentum of Light and the Transformation of Laguerre-Gaussian Laser Modes. Physical Review A.

[28] Albaladejo S, Marques MI, Scheffold F, Saenz JJ (2009). Giant Enhanced Diffusion of Gold Nanoparticles in Optical Vortex Fields. Nano Lett..

[29] Curtis JE, Grier DG (2003). Structure of optical vortices. Physical Review Letters.

[30] Ng J, Lin ZF, Chan CT (2010). Theory of Optical Trapping by an Optical Vortex Beam. Physical Review Letters.

[31] Khan M, Sood AK, Deepak FL, Rao CNR (2006). Nanorotors using asymmetric inorganic nanorods in an optical trap. Nanotechnology.

[32] Ding, K., Ng, J., Zhou, L. & Chan, C. T. Realization of optical pulling forces using chirality. *Physical Review A* **89**10.1103/PhysRevA.89.063825 (2014).

[33] Hakobyan D, Brasselet E (2014). Left-handed optical radiation torque. Nature Photonics.

[34] Wang, S. B. & Chan, C. T. Lateral optical force on chiral particles near a surface. *Nat. Commun*. 5, doi:ARTN 3307 10.1038/ncomms4307 (2014). 10.1038/ncomms4307 PMC3959198 24598792

[35] Chaumet PC, Rahmani A (2009). Electromagnetic force and torque on magnetic and negative-index scatterers. Optics Express.

[36] Wulff KD, Cole DG, Clark RL (2008). Controlled rotation of birefringent particles in an optical trap. Appl. Optics.

[37] Chang S, Lee SS (1985). Optical Torque Exerted on a Homogeneous Sphere Levitated in the Circularly Polarized Fundamental-Mode Laser-Beam. J Opt Soc Am B.

[38] Williams I (2016). Transmission of torque at the nanoscale. Nat. Phys..

[39] Nieto-Vesperinas M (2015). Optical torque on small bi-isotropic particles. Optics Letters.

[40] Barton JP, Alexander DR, Schaub SA (1989). Theoretical Determination of Net-Radiation Force and Torque for a Spherical-Particle Illuminated by a Focused Laser-Beam. Journal of Applied Physics.

[41] Geng Y (2016). Flexible optical manipulation of ring resonator by double port excitation. Opt Express.

[42] Cao T, Mao LB, Gao DL, Ding WQ, Qiu CW (2016). Fano resonant Ge2Sb2Te5 nanoparticles realize switchable lateral optical force. Nanoscale.

[43] Kanjanasit K, Wang CH (2013). Fano resonance in a metamaterial consisting of two identical arrays of square metallic patch elements separated by a dielectric spacer. Applied Physics Letters.

[44] Liaw JW, Lo WJ, Lin WC, Kuo MK (2015). Theoretical study of optical torques for aligning Ag nanorods and nanowires. J. Quant. Spectrosc. Radiat. Transf..

[45] Katz DS, Thiele ET, Taflove A (1994). Validation and extension to 3 dimensions of the berenger PML absorbing boundary-condition for FD-TD meshes. IEEE Microw. Guided Wave Lett..

